# U-Shaped Association of Visceral Fat Area With Diabetic Peripheral Neuropathy

**DOI:** 10.1155/jdr/3291418

**Published:** 2025-05-18

**Authors:** Xianhua Li, Yingxiang Wang, Xiaotong Feng, Lin Mao, Jing Ke, Dong Zhao

**Affiliations:** Center for Endocrine Metabolism and Immune Diseases, Beijing Luhe Hospital Affiliated to Capital Medical University, Beijing, China

**Keywords:** cross-sectional study, diabetic peripheral neuropathy, type 2 diabetes mellitus, visceral fat area

## Abstract

**Background:** The impact of visceral fat area (VFA) on diabetic peripheral neuropathy (DPN) remains controversial in Type 2 diabetes mellitus (T2DM), with conflicting evidence.

**Methods:** We conducted a cross-sectional study at the National Metabolic Management Center of Beijing Luhe Hospital between October 2017 and May 2024. VFA was quantified using bioelectrical impedance analysis, and DPN was diagnosed according to standardized clinical criteria. The association between VFA and DPN was examined using multiple logistic regression models with comprehensive confounder adjustment. Nonlinear relationships were investigated through generalized additive models and threshold effect analyses.

**Results:** Among 7436 T2DM patients (3044 females), the median VFA was 104 cm^2^ (interquartile range: 79.5–132 cm^2^), with DPN present in 26.55% of participants. Generalized additive models revealed a significant U-shaped association between VFA and DPN, with an inflection point at 133 cm^2^ (log-likelihood ratio test, *p* < 0.001). In the segmented regression analysis, each 10 cm^2^ increase in VFA below this threshold was associated with decreased DPN risk (OR: 0.95, 95% CI: 0.93–0.97, *p* < 0.05), while increases above the threshold were associated with elevated risk (OR: 1.06, 95% CI: 1.03–1.09, *p* < 0.05).

**Conclusions:** A U-shaped association exists between VFA and DPN prevalence in T2DM patients, suggesting clinical relevance of moderate visceral adiposity.

## 1. Introduction

Diabetic peripheral neuropathy (DPN) is a prevalent complication of diabetes mellitus, affecting up to 50% of individuals with diabetes over their lifetime [1]. This condition is associated with significant disability, compromised quality of life, and increased mortality [2, 3]. Despite its substantial clinical burden, the pathophysiological mechanisms underlying DPN remain incompletely understood [4]. Emerging evidence suggests a complex relationship between visceral adiposity and diabetic complications, including DPN [5, 6]. Visceral fat tissue, characterized by its location surrounding internal organs, functions as an active endocrine organ, secreting various adipokines and proinflammatory cytokines [7–10]. These bioactive substances have been implicated in multiple pathological processes, including insulin resistance, oxidative stress, and chronic low-grade inflammation, which may contribute to peripheral nerve dysfunction in diabetes [11–13].

The quantification of visceral fat area (VFA) has emerged as a precise metric for metabolic assessment, potentially offering advantages over conventional anthropometric measurements such as body mass index or waist circumference [14, 15]. Bioelectrical impedance analysis (BIA) provides a noninvasive and accessible method for VFA estimation, facilitating comprehensive investigation of its clinical associations [16, 17]. Previous studies examining the association between VFA and DPN have yielded inconsistent results [5, 18]. We postulate that these discrepancies might be explained by potential nonlinear relationships between these variables, an aspect not adequately addressed in existing literature. This investigation aims to characterize the relationship between BIA-estimated VFA and DPN prevalence in patients with Type 2 diabetes mellitus (T2DM). Our findings may enhance the understanding of the metabolic factors associated with diabetic neuropathy.

## 2. Methods

### 2.1. Study Design and Population

This cross-sectional investigation was conducted at the National Metabolic Management Center (MMC) of Beijing Luhe Hospital between October 27, 2017, and May 17, 2024. The MMC, a nationwide initiative, implements standardized management protocols for metabolic disorders [19]. Participants underwent comprehensive evaluations, including laboratory testing, systematic physical examinations, and structured questionnaire interviews. The study population comprised adults aged 18 years or older with T2DM. T2DM diagnosis was established according to the 1999 World Health Organization criteria, defined as either fasting plasma glucose ≥ 7.0 mmol/L, 2-h post-load glucose ≥ 11.1 mmol/L during an oral glucose tolerance test, or a documented physician diagnosis based on these criteria.

**Figure 1:**
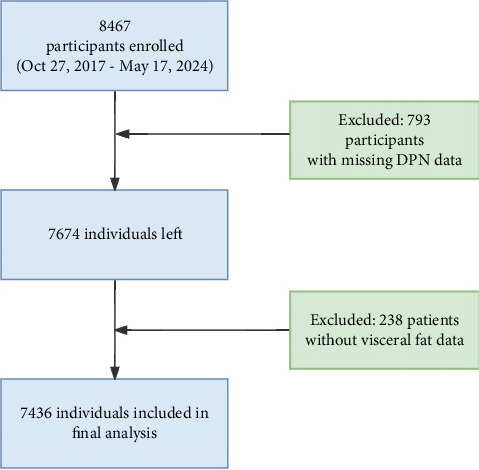
Flow diagram of study participant selection.

We excluded participants with other types of diabetes mellitus, severe diabetic foot ulcers (Wagner classification Grades III–V), or previous amputation. Additional exclusion criteria included recent acute diabetic complications, nondiabetic neuropathies, severe respiratory disease, advanced heart failure, and hematological or thromboembolic disorders. Participants were also excluded if they were pregnant or lactating, using medications that could affect peripheral nerve function or the sympathetic nervous system (including immunosuppressants, antioxidants, anti-inflammatories, antibiotics, analgesics, systemic corticosteroids, and certain vitamins or supplements), or had recent significant weight changes or a history of bariatric surgery. Of the 8467 patients initially screened, 1031 were excluded due to missing data (793 for DPN assessment and 238 for visceral fat measurements), resulting in a final analytical sample of 7436 participants. A detailed flow diagram of the study population selection process is presented in Figure 1.

### 2.2. Ethical Considerations

This study was conducted in accordance with the Declaration of Helsinki. The protocol was reviewed and approved by the Medical Ethics Committee of Beijing Luhe Hospital, Capital Medical University (Approval Number: 2023-LHKY-094-02).

### 2.3. Data Collection and Measurements

Demographic and clinical information was obtained through standardized questionnaires and electronic medical records. Lifestyle factors were systematically categorized: alcohol consumption as nondrinker, occasional drinker (< weekly), or regular drinker (≥ weekly) and smoking status as nonsmoker, occasional smoker (< daily or < 7 cigarettes/week), or regular smoker (daily). Anthropometric measurements followed standardized protocols. BMI was calculated as weight (kg)/height (m)^2^. BMI was categorized according to Chinese-specific criteria as recommended by the Working Group on Obesity in China [20]. Blood pressure was measured in triplicate using calibrated sphygmomanometers after a 5-min seated rest, averaging the latter two readings. Blood samples were collected after ≥ 8 h fasting. Biochemical parameters (FPG, LDL-C, HDL-C, and TG) were analyzed using an automated analyzer (AU5800, Beckman Coulter, United States). HbA1c was determined by HPLC (D10 analyzer, Bio-Rad, United States). Hypertension was defined as systolic blood pressure ≥ 140 mmHg, diastolic blood pressure ≥ 90 mmHg, or antihypertensive medication use [21]. Hyperlipidemia was identified through lipid profile abnormalities or lipid-lowering medication use [22]. Diabetic retinopathy was assessed via standardized two-field fundus photography and classified according to the International Classification of Diabetic Retinopathy scale [23]. Antidiabetic medication use was documented through physician interviews, recording patients' current medications including SGLT-2 inhibitors, GLP-1 receptor agonists, biguanides, sulfonylureas, and insulin.

### 2.4. VFA Assessment

VFA was quantified using dual BIA with the HDS 2000 system (Omron Co. Ltd., Kyoto, Japan). Measurements were performed by trained researchers following standardized protocols. After an 8-h fast, participants were positioned supine with exposed ankles, wrists, and abdomen. Electrode detectors were placed at these sites, and an electrode belt was secured around the abdomen. Participants maintained calm breathing and briefly suspended respiration at end-expiration during measurement. The system first measured the horizontal umbilical abdominal cross-sectional area, then automatically calculated VFA in square centimeters. Measurements were repeated if any technical deviations occurred to ensure data quality.

### 2.5. DPN Assessment

The evaluation of DPN followed a systematic approach. Initial clinical assessment included detailed history-taking of neuropathic symptoms, encompassing numbness, pain (characterized as prickling, stabbing, shooting, burning, or aching), and paresthesia (including abnormal temperature sensation, allodynia, and hyperalgesia) in the extremities [24]. Neurological examinations were conducted by experienced physicians, evaluating vibration perception, light touch sensitivity, and bilateral Achilles tendon reflexes in the knee-standing position.

Quantitative assessment was performed using the DPN Check device (Neuro Metrix Inc., Waltham, MA, United States), an FDA-approved point-of-care nerve conduction system. This method was selected to enhance diagnostic objectivity compared to symptom-based assessments. The device evaluates sural nerve conduction velocity (CV) and amplitude. Following standardized skin preparation, biosensor probes were positioned on the lateral lower leg, proximal to the ankle. Sural nerve responses were elicited by electrical stimulation and detected by the biosensor. Three measurements were obtained for each leg, with peak CV and amplitude values recorded. The DPN Check system has demonstrated robust diagnostic performance when validated against conventional nerve conduction studies [25]. DPN diagnosis integrated DPN Check results with clinical symptoms and neurological examination findings [26].

### 2.6. Statistical Analysis

Continuous variables were expressed as mean ± standard deviation (SD) for normally distributed data or median (interquartile range) for skewed distributions. Categorical variables were presented as frequencies and percentages. Between-group comparisons across VFA quartiles employed one-way ANOVA for normally distributed continuous variables, Kruskal–Wallis *H* test for skewed continuous variables, and chi-square test for categorical variables.

The association between VFA and DPN was examined using multiple logistic regression models with confounder adjustment. The crude model included no covariates. Model I adjusted for demographic factors (age and sex). Model II further adjusted for diabetes-related parameters and lifestyle factors (diabetes duration, fasting blood glucose, HbA1c, smoking status, alcohol consumption, and eGFR). Model III additionally incorporated comorbidities (hypertension, hyperlipidemia, and diabetic retinopathy).

Potential nonlinear relationships were investigated using generalized additive models with penalized splines. Upon detection of nonlinearity, a two-piecewise logistic regression model was fitted with the optimal inflection point identified by recursive algorithm. The log-likelihood ratio test was employed to compare the two-piecewise versus linear models. In sensitivity analyses, we further adjusted for these antidiabetic medications in generalized additive models.

We performed statistical analyses using R software (Version 4.2.0, R Foundation for Statistical Computing, Vienna, Austria) and EmpowerStats (X&Y Solutions, Inc., Boston, MA, United States). A two-sided *p* value < 0.05 was considered statistically significant.

## 3. Results

Table 1 presents the baseline characteristics of 7436 participants stratified by VFA quartiles. Significant interquartile differences were observed in most characteristics. Higher VFA quartiles were characterized by younger age, male predominance, and increased BMI. The proportion of regular alcohol consumption and smoking demonstrated an ascending trend across quartiles. Metabolic parameters, including FPG and HbA1c levels, showed progressive elevation with increasing VFA. However, median diabetes duration exhibited an inverse relationship. The prevalence of hypertension and hyperlipidemia increased substantially across VFA quartiles. Notably, DPN prevalence showed a nonlinear distribution, with the highest rate observed in Q1 (30.55%), followed by relatively lower rates in subsequent quartiles (*p* < 0.001). Antidiabetic medication use varied across VFA quartiles. The use of SGLT-2 inhibitors increased from Q1 to Q4, as did GLP-1 receptor agonists and biguanides. Sulfonylureas showed higher use in middle quartiles and lowest in Q4. Insulin use remained relatively consistent across quartiles (*p* = 0.318).

Table 2 presents the association between VFA and DPN across different adjustment models. When analyzing VFA as a continuous variable, each 10 cm^2^ increase in VFA was inversely associated with DPN risk in the crude model (OR: 0.98, 95% CI: 0.97–0.99, *p* < 0.01). This association persisted after adjusting for demographic factors and metabolic parameters (Models I and II) but was attenuated to nonsignificance in the fully adjusted model (Model III: OR: 0.99, 95% CI: 0.98–1.00, *p* = 0.14). In the quartile analysis, compared with Q1, all other quartiles showed significantly lower DPN risk. This pattern remained consistent across all adjustment models, with Q3 demonstrating the strongest protective association. In the fully adjusted model (Model III), the odds ratios for Q2, Q3, and Q4 were 0.85 (95% CI: 0.72–1.00, *p* = 0.05), 0.79 (95% CI: 0.67–0.93, *p* < 0.01), and 0.82 (95% CI: 0.69–0.97, *p* = 0.02), respectively, suggesting a nonlinear relationship between the VFA and DPN risk.

**Figure 2:**
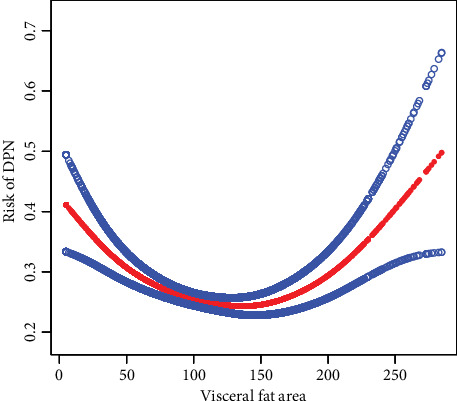
Nonlinear association between visceral fat area and risk of diabetic peripheral neuropathy using generalized additive model. Figure annotation: The generalized additive model (GAM) plot shows the relationship between visceral fat area (VFA) and risk of diabetic peripheral neuropathy (DPN). The red line represents the fitted curve with blue shaded area indicating 95% confidence interval. The model was adjusted for age, sex, smoking status, alcohol consumption, fasting blood glucose (FBG), glycated hemoglobin (HbA1c), estimated glomerular filtration rate (eGFR), diabetes duration, hypertension, hyperlipidemia, and diabetic retinopathy.

Figure 2 illustrates a U-shaped relationship between VFA and the risk of DPN after adjusting for potential confounders. The curve demonstrates that the risk of DPN initially decreases with increasing VFA until reaching a nadir at moderate VFA levels, followed by a gradual increase in risk with further VFA elevation. The blue dots representing the 95% confidence intervals support the statistical significance of this nonlinear association. This U-shaped pattern suggests that both extremely low and high VFA values may be associated with increased DPN risk, while moderate VFA levels correspond to the lowest risk. In sensitivity analyses, we further adjusted for key antidiabetic medications including SGLT-2 inhibitors, GLP-1 receptor agonists, biguanides, sulfonylureas, and insulin. Our results demonstrate that even after adjusting for these medications, the U-shaped association between VFA and DPN persisted.

Table 3 presents the threshold effect analysis of VFA on DPN risk. The nonlinear model (Model II) identified a significant threshold effect at 133 cm^2^ of VFA. Below this threshold, each 10 cm^2^ increase in VFA was associated with a 5% decreased risk of DPN (OR: 0.95, 95% CI: 0.93–0.97, *p* < 0.001). Conversely, above the threshold, each 10 cm^2^ increase in VFA was associated with a 6% increased risk of DPN (OR: 1.06, 95% CI: 1.03–1.09, *p* < 0.001). To translate these findings into clinical context, for patients with low visceral adiposity (VFA < 133 cm^2^), an increase of 10 cm^2^ in VFA would be associated with approximately 5% lower DPN risk. Conversely, for patients with higher visceral adiposity (VFA > 133 cm^2^), an increase of 10 cm^2^ would be associated with approximately 6% higher DPN risk. The log-likelihood ratio test (*p* < 0.001) confirmed that the nonlinear model fitted the data significantly better than the linear model.

## 4. Discussion

This study reveals a significant U-shaped relationship between VFA and DPN. This finding adds a new dimension to our understanding of the complex interplay between visceral adiposity and diabetic complications.

Our findings both complement and extend previous research on the VFA-DPN association. Wu et al., in a study of 2498 hospitalized patients with Type 2 diabetes, reported that the DPN group had significantly lower VFA than the non-DPN group, with VFA negatively correlated with DPN presence (*p* < 0.001) [18]. Conversely, Oh et al. observed higher VFA in DPN patients, demonstrating a positive association (OR: 1.026; 95% CI: 1.005–1.048; *p* = 0.015) after confounder adjustment [5]. These previous studies likely missed the U-shaped relationship due to smaller sample sizes, use of linear regression models that assume monotonic relationships, and patient populations possibly clustered at different points along the VFA spectrum. Our larger study population and specific application of nonlinear modeling techniques enabled detection of this more complex association pattern.

The U-shaped relationship between VFA and DPN (inflection point: 133 cm^2^) has important clinical implications. Clinicians should consider both very low and high VFA as associated with increased DPN risk, beyond the typical obesity focus. Patients with very low or very high VFA warrant closer neurological monitoring as they might be overlooked. This threshold offers a reference for risk stratification, though different samples and studies are needed to validate this specific cutoff point. Including VFA as a nonlinear parameter in risk assessment tools could improve their performance compared to models using only traditional factors.

Distinct physiological mechanisms at different VFA levels may explain the observed U-shaped relationship. At lower VFA levels, modest increases may be associated with metabolic benefits, potentially through enhanced secretion of beneficial adipokines or improved insulin sensitivity [27–29]. This protective effect may also relate to fat distribution patterns, where appropriate visceral adiposity could prevent ectopic fat deposition in the liver, muscle, and pancreas [30]. Additionally, the balance between subcutaneous (more metabolically benign) and visceral fat likely influences this relationship, with proportional increases in subcutaneous fat potentially offering neuroprotective effects through favorable adipokine profiles [31, 32]. Conversely, excessive visceral adiposity beyond the identified threshold might be associated with DPN through various pathways, including chronic low-grade inflammation, oxidative stress, and insulin resistance [8, 33, 34]. While our study identifies a U-shaped association between VFA and DPN in humans, this relationship has not been directly examined in animal models or isolated tissues. Future studies designed to test this U-shaped hypothesis across different experimental models could provide valuable mechanistic insights and potentially identify new therapeutic targets for DPN management.

Several strengths characterize our study. The utilization of the National MMC dataset [19] provided standardized VFA measurements and precise DPN assessments across a substantial sample size. Our analytical approach, incorporating smooth curve fitting and threshold effect analysis, enabled detailed examination of the VFA–DPN relationship. The comprehensive adjustment strategy enhanced the robustness of our findings. Nevertheless, important limitations merit consideration. The cross-sectional design precludes causal inference, necessitating longitudinal studies to examine temporal relationships between VFA changes and DPN. Despite extensive adjustment, residual confounding cannot be excluded. Additionally, given the epidemiological variations in obesity across different populations [35], our findings may have limited generalizability to populations distinct from our study. Another limitation of our study is the use of eGFR without assessment of muscle mass. Since serum creatinine is influenced by muscle mass, eGFR calculations may overestimate kidney function in patients with reduced muscle mass, which is common in diabetic populations. Future studies should consider incorporating muscle mass measurements or using alternative kidney function markers less affected by muscle mass, such as cystatin C-based eGFR equations. Another limitation of our study is the use of BIA rather than imaging methods like CT or MRI for VFA assessment. While BIA offers practical advantages in large-scale studies, including lower cost, convenience, and absence of radiation exposure, it may be influenced by hydration status and other physiological variables. Studies comparing BIA with CT-derived VFA measurements have shown strong correlation though BIA may underestimate VFA in severely obese individuals [36, 37]. Future studies using imaging-based VFA quantification would be valuable to confirm the observed U-shaped relationship.

## 5. Conclusion

Our study demonstrates a U-shaped association between VFA and DPN prevalence in Type 2 diabetes patients. Future research should include interventional studies to investigate whether targeted VFA modulation through weight management or pharmacological approaches can influence DPN outcomes. Additionally, longitudinal studies are needed to establish temporal relationships between VFA changes and DPN progression. These complementary approaches would strengthen evidence for causality and guide preventive strategies targeting visceral adiposity in diabetic neuropathy management.

## Figures and Tables

**Table 1:** Baseline characteristics of study population stratified by visceral fat area.

	**Q1 (5.00–79.40)**	**Q2 (79.50–103.90)**	**Q3 (104.00–131.90)**	**Q4 (132.00–386.00)**	**p** ** value**
Age (years)	51.75 (12.18)	52.79 (12.16)	51.62 (12.18)	49.67 (13.42)	< 0.001
Sex, *n* (%)					< 0.001
Female	1002 (53.99%)	869 (48.06%)	675 (35.75%)	498 (26.43%)	
Male	854 (46.01%)	939 (51.94%)	1213 (64.25%)	1386 (73.57%)	
BMI (kg/m^2^), *n* (%)					< 0.001
≤ 24	1092 (59.03%)	437 (24.21%)	134 (7.11%)	37 (1.97%)	
> 24, ≤ 28	685 (37.03%)	1080 (59.83%)	1016 (53.90%)	391 (20.81%)	
> 28	73 (3.95%)	288 (15.96%)	735 (38.99%)	1451 (77.22%)	
Smoking status, *n* (%)					< 0.001
Nonsmoker	1375 (74.36%)	1279 (70.86%)	1194 (63.31%)	1097 (58.41%)	
Occasional smoker	67 (3.62%)	63 (3.49%)	108 (5.73%)	117 (6.23%)	
Regular smoker	407 (22.01%)	463 (25.65%)	584 (30.97%)	664 (35.36%)	
Alcohol consumption, *n* (%)					< 0.001
Nondrinker	1329 (71.92%)	1166 (64.60%)	1034 (54.83%)	956 (50.91%)	
Occasional drinker	391 (21.16%)	460 (25.48%)	586 (31.07%)	639 (34.03%)	
Regular drinker	128 (6.93%)	179 (9.92%)	266 (14.10%)	283 (15.07%)	
FPG (mmol/L)	8.71 (3.82)	9.08 (3.64)	9.17 (3.49)	9.47 (3.81)	< 0.001
HBA1C (%)	8.80 (2.51)	8.75 (2.18)	8.84 (2.05)	9.10 (2.04)	< 0.001
eGFR (mL/(min × 1.73 m^2^))	103.43 (16.93)	101.13 (17.58)	102.31 (17.64)	103.01 (19.37)	< 0.001
Diabetes duration (months)	65.00 (9.00–136.00)	63.00 (6.00–140.00)	51.00 (4.00–128.00)	36.00 (1.00–119.00)	< 0.001
Diabetic retinopathy, *n* (%)					0.002
No	1269 (70.74%)	1269 (72.97%)	1372 (75.63%)	1358 (75.32%)	
Yes	525 (29.26%)	470 (27.03%)	442 (24.37%)	445 (24.68%)	
Hypertension *n* (%)					< 0.001
No	1310 (70.89%)	1066 (59.12%)	1037 (55.07%)	873 (46.44%)	
Yes	538 (29.11%)	737 (40.88%)	846 (44.93%)	1007 (53.56%)	
Hyperlipidemia, *n* (%)					< 0.001
No	1288 (69.70%)	1065 (59.07%)	1038 (55.10%)	957 (50.93%)	
Yes	560 (30.30%)	738 (40.93%)	846 (44.90%)	922 (49.07%)	
Diabetic peripheral neuropathy, *n* (%)					< 0.001
No	1289 (69.45%)	1339 (74.06%)	1432 (75.85%)	1402 (74.42%)	
Yes	567 (30.55%)	469 (25.94%)	456 (24.15%)	482 (25.58%)	
Antidiabetic medication use					
SGLT-2 inhibitors	64 (3.49%)	87 (4.86%)	83 (4.43%)	119 (6.38%)	< 0.001
GLP-1 receptor agonists	7 (0.38%)	17 (0.95%)	46 (2.46%)	74 (3.97%)	< 0.001
Biguanides	702 (38.32%)	812 (45.39%)	889 (47.46%)	919 (49.28%)	< 0.001
Sulfonylureas	180 (9.83%)	206 (11.51%)	187 (9.98%)	130 (6.97%)	<0.001
Insulin, *n* (%)	309 (16.94%)	337 (18.90%)	321 (17.19%)	313 (16.83%)	0.318

*Note*: Data are presented as mean ± standard deviation for normally distributed continuous variables, median (interquartile range) for nonnormally distributed continuous variables, and *n* (%) for categorical variables.

**Table 2:** Association between visceral fat area and diabetic peripheral neuropathy in different adjustment models.

**Exposure**	**Crude model**	**Model I**	**Model II**	**Model III**
Visceral fat area (per 10 cm^2^ increase)	0.98 (0.97, 0.99) < 0.01	0.97 (0.96, 0.99) < 0.01	0.98 (0.96, 0.99) < 0.01	0.99 (0.98, 1.00) 0.14
Visceral fat area subgroups				
Q1	1.0	1.0	1.0	1.0
Q2	0.80 (0.69, 0.92) < 0.01	0.78 (0.67, 0.90) 0.01	0.79 (0.67, 0.92) < 0.01	0.85 (0.72, 1.00) 0.05
Q3	0.72 (0.63, 0.84) < 0.01	0.68 (0.59, 0.79) < 0.01	0.70 (0.60, 0.82) < 0.01	0.79 (0.67, 0.93) < 0.01
Q4	0.78 (0.68, 0.90) < 0.01	0.72 (0.62, 0.84) < 0.01	0.71 (0.61, 0.83) < 0.01	0.82 (0.69, 0.97) 0.02

*Note*: Data are presented as odds ratio (OR) with 95% confidence interval (CI) and *p* value. Models: crude model: no covariates adjusted; Model I: adjusted for age and sex; Model II: adjusted for age, sex, smoking status, alcohol consumption, fasting blood glucose (FBG), glycated hemoglobin (HbA1c), estimated glomerular filtration rate (eGFR), and diabetes duration; Model III: adjusted for age, sex, smoking status, alcohol consumption, FBG, HbA1c, eGFR, diabetes duration, hypertension, hyperlipidemia, and diabetic retinopathy.

**Table 3:** Threshold effect analysis of visceral fat area on diabetic peripheral neuropathy (DPN).

**Model**	**OR (95% CI)**	**p** ** value**
Model I (linear effect)		
One-line effect	0.99 (0.98, 1.00)	0.14
Model II (nonlinear effect)		
Threshold (cm^2^)	133	
Effect for < 133	0.95 (0.93, 0.97)	< 0.001
Effect for > 133	1.06 (1.03, 1.09)	< 0.001
Log-likelihood ratio test		< 0.001

*Note*: Exposure: visceral fat area (per 10 cm^2^ increase). Outcome: DPN. Adjusted for: age, sex, smoking status, alcohol consumption, FBG, HbA1c, eGFR, diabetes duration, hypertension, hyperlipidemia, and diabetic retinopathy. *p* < 0.001 for log-likelihood ratio test indicates that the nonlinear model (Model II) is significantly different from the linear model (Model I).

Abbreviations: CI = confidence interval; OR = odds ratio.

## Data Availability

The data that support the findings of this study are available from the corresponding author upon reasonable request.
